# High-quality sandwiched black phosphorus heterostructure and its quantum oscillations

**DOI:** 10.1038/ncomms8315

**Published:** 2015-06-23

**Authors:** Xiaolong Chen, Yingying Wu, Zefei Wu, Yu Han, Shuigang Xu, Lin Wang, Weiguang Ye, Tianyi Han, Yuheng He, Yuan Cai, Ning Wang

**Affiliations:** 1 Department of Physics and the William Mong Institute of Nano Science and Technology, The Hong Kong University of Science and Technology, Clear Water Bay, Hong Kong, China; 2 Department of Condensed Matter Physics, Group of Applied Physics, University of Geneva, 24 Quai Ernest Ansermet, CH1211 Geneva, Switzerland

## Abstract

Two-dimensional materials such as graphene and transition metal dichalcogenides have attracted great attention because of their rich physics and potential applications in next-generation nanoelectronic devices. The family of two-dimensional materials was recently joined by atomically thin black phosphorus which possesses high theoretical mobility and tunable bandgap structure. However, degradation of properties under atmospheric conditions and high-density charge traps in black phosphorus have largely limited its actual mobility thus hindering its future applications. Here, we report the fabrication of stable sandwiched heterostructures by encapsulating atomically thin black phosphorus between hexagonal boron nitride layers to realize ultra-clean interfaces that allow a high field-effect mobility of ∼1,350 cm^2^V^−1^ s^−1^ at room temperature and on–off ratios exceeding 10^5^. At low temperatures, the mobility even reaches ∼2,700 cm^2^V^−1^ s^−1^ and quantum oscillations in black phosphorus two-dimensional hole gas are observed at low magnetic fields. Importantly, the sandwiched heterostructures ensure that the quality of black phosphorus remains high under ambient conditions.

## Abstract (web summary)

Black phosphorus is an atomically thin material that exhibits excellent properties for electronics applications, but these degrade in atmospheric conditions. Here, the authors demonstrate the fabrication of stable, ultra-clean and high-mobility black phosphorus sandwiched between the layers of boron nitride.

## Graphical abstract

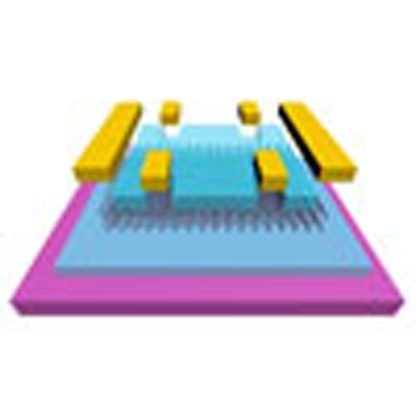

Two-dimensional (2D) materials with both high mobility and high on–off ratios are in demand for next-generation nanodevices. Graphene^1^,^2^, being the most widely studied material, has shown rich physics and high mobility, but the absence of a bandgap^1^,^2^ limits its applications. Analogous to 2D transition metal dichalcogenide structures^3^,^4^,^5^,^6^,^7^,^8^,^9^,^10^,^11^, atomically thin black phosphorus (BP)^12^,^13^,^14^,^15^ has recently evoked interest due to its high theoretical mobility^16^, tunable direct bandgap^17^,^18^,^19^,^20^ and ambipolarity^12^,^13^,^14^,^15^—unique properties that make it suitable for application in electronic and optoelectronic devices^21^,^22^,^23^. Recently, field-effect transistors (FETs)^12^,^13^,^14^,^15^,^24^,^25^,^26^, electrically tunable PN junctions^22^, radio-frequency transistors^27^ and heterojunctions^28^,^29^ based on few-layer BP have also been demonstrated. However, the room temperature mobility of few-layer phosphorene reported so far is limited to 400 cm^2^V^−1^ s^−1^ due to the presence of high-density charge traps, defects and phonon scattering^12^,^13^,^14^,^15^. The quality degradation of BP under atmospheric conditions is mainly due to its reaction with O_2_-saturated H_2_O^30^,^31^.

Here we demonstrate a high field-effect mobility of ∼1,350 cm^2^V^−1^ s^−1^ at room temperature and high on–off ratios exceeding 10^5^ in few-layer phosphorene encapsulated by atomically thin hexagonal boron nitride (BN), which forms a stable BN–BP–BN heterostructure. A room temperature mobility of ∼1,000 cm^2^V^−1^ s^−1^ is rare in 2D semiconductor electron gas systems prepared by mechanical exfoliation techniques. In addition, the BN layers further avoids quality degradation of BP when exposed to atmosphere. At cryogenic temperatures, the mobility even reaches ∼2,700 cm^2^V^−1^ s^−1^ and Shubnikov-de Haas (SdH) oscillations are observed in BP at low magnetic fields (∼6 T). Our fabrication and treatment techniques for BN–BP–BN heterostructures open up a way to achieve high-performance 2D semiconductors with greatly improved room temperature mobility, on–off ratios and stability under ambient conditions for practical applications in high-speed electronic and optoelectronic devices.

## Results

### Fabrication of the sandwiched BP heterostructure devices

**Figure 1:**
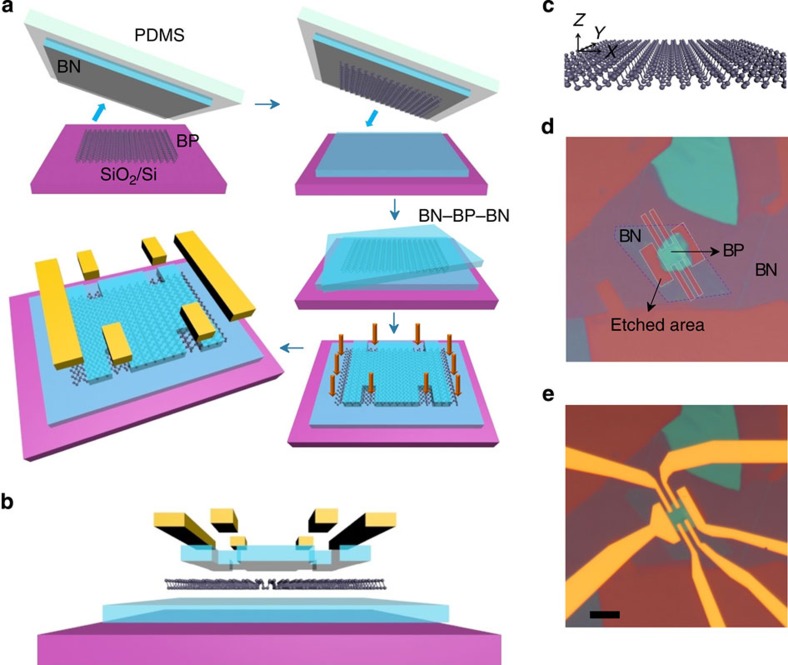
BN–BP–BN heterostructure device. (**a**) Schematic of the BN–BP–BN heterostructure device fabrication process. (**b**,**e**) Schematic (**b**) and optical image (**e**) of a BN–BP–BN Hall-bar device. Scale bar, 10 μm. (**c**) Atomic structure of monolayer BP. (**d**) The BN–BP–BN heterostructure after O_2_-plasma etching. The etched area is enclosed within the white line. The purple-dashed line denotes the lower BN layer.

**Figure 2:**
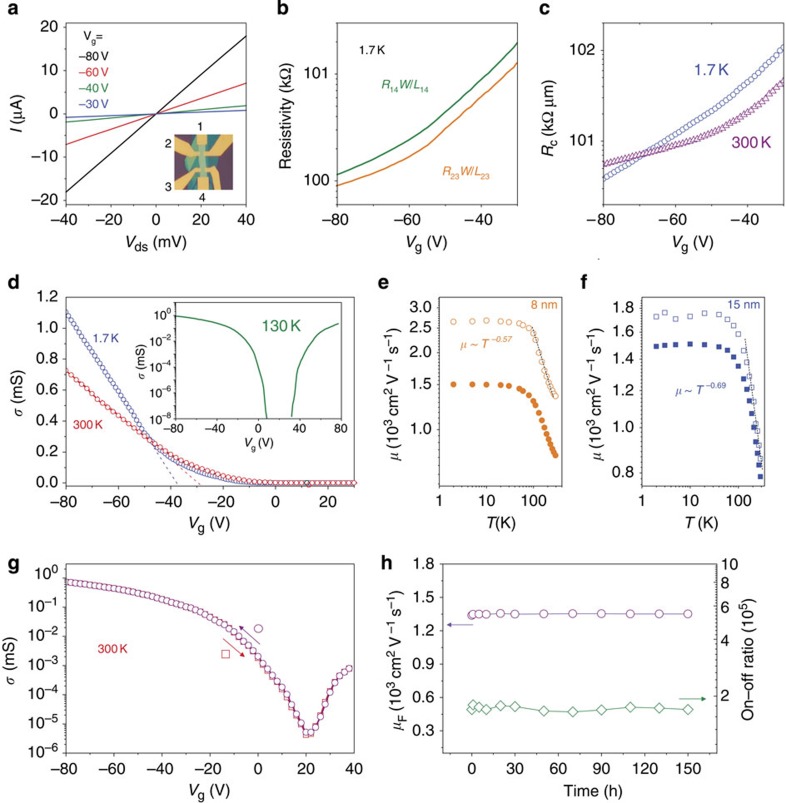
Mobility and stability of the BN–BP–BN heterostructure devices. (**a**) The *I*–*V*_ds_ curves obtained at different gate voltages at 1.7 K. The inset is the optical image of Sample A with the following geometrical parameters: *L*_14_=16 μm, *L*_23_=10 μm and *W*=3 μm. (**b**) The resistivity determined from four-terminal (green line) and two-terminal configurations (orange line) at 1.7 K. (**c**) Variation of the contact resistivity. (**d**) The conductivity of Sample A measured at with a room temperature and 1.7 K. The inset shows the ambipolarity of the BP conductance. (**e**,**f**) Temperature dependence of the field-effect *μ*_F_ (open dots) and Hall mobility*μ*_h_ (solid dots) at *V*_g_=−70 V) of Sample A and Sample B (15 nm thick). The dashed lines serve as guidelines for the *μ*∼*T*^−*γ*^ relation. (**g**) The room temperature conductivity showing no hysteresis in Sample A. (**h**) The mobility and on–off ratio of Sample A as a functions of ambient exposure time. No quality degradation is observed even after exposure for an entire week.

To achieve both high mobility and stability of BP FETs under atmospheric conditions, the BN–BP–BN configuration and high-temperature annealing are the two key factors. The ultra-clean BN–BP interfaces are ensured by adopting the polymer-free van der Waals transfer technique^32^ as shown in Fig. 1a. The few-layer BP mechanically exfoliated on a 300-nm SiO_2_/Si substrate was first picked up by a thin BN flake (6–20 nm thick). Then the BN–BP sample was transferred to a BN flake supported on a SiO_2_/Si substrate to form the BN–BP–BN heterostructure. The atomically thin BP was completely encapsulated by two BN layers, thus allowing us to anneal the sample at temperatures up to 500 °C in an argon atmosphere to further improve the sample quality. Without the BN protective layers, few-layer BP breaks down easily at 350 °C (see Supplementary Note 1 and Supplementary Fig. 1). In addition, the annealing process can significantly reduce the charge trap density in BP as no hysteresis effect is observed at room temperature (Fig. 2g and Supplementary Fig. 2).

Instead of using one-dimensional edge-contact^32^ that has been proven effective for graphene-encapsulated structures (but less so for 2D semiconductors), we developed the area contact technique for contacting the encapsulated 2D semiconductors. Beginning with the BN–BP–BN heterostructure, a hard mask is defined by the standard electron-beam lithography technique using ZEP-520 resist (detailed process is shown in Supplementary Note 2 and Supplementary Fig. 3). Since the O_2_-plasma etching rates for BN and BP are different, the BN layers can be quickly etched while the BP layer still survive as shown in Fig. 1d. Lastly, Cr/Au (2 nm/60 nm) electrodes are deposited using electron-beam lithography technique. Figure 1b,e show the schematic and optical image of a BN–BP–BN Hall-bar device respectively. Note that no further annealing process is needed after deposition of the electrodes.

### Mobility and stability of the sandwiched BP devices

Figure 2a shows the *I*–*V*_ds_ characteristic of an 8-nm-thick BN–BP–BN sample (Sample A). The linear *I*–*V*_ds_ characteristic indicates that Ohmic contacts have been achieved on the hole side. By comparing the resistivity directly measured using the two-terminal configuration (*R*_14_*W*/*L*_14_) with that extracted using the four-terminal configuration (*R*_23_*W*/*L*_23_), the contact resistivity is obtained (Fig. 2b). As shown in Fig. 2c, the contact resistivity measured at high gate voltages is about several 
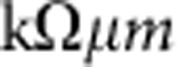
 when the sample temperature is sufficiently low and thermal activation effects are suppressed. The contact resistivity decreases with decreasing temperature when the gate voltage *V*_g_<−68 V, further verifying the good contacts between BP and Cr/Au electrodes on the hole side.

The BN–BP–BN heterostructure exhibits high quality as is first confirmed by examining the four-terminal field-effect (FET) mobility 
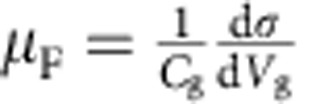
 at different temperatures, where *σ* is the conductivity and *C*_g_ is the gate capacitance. Figure 2d shows the conductivity as a function of gate voltage for Sample A. Similar to the results reported previously, both hole and electron conductance can be achieved (see the inset in Fig. 2d). Here we mainly focus on the hole conductance due to its higher mobility. The gate capacitance *C*_g_ is calculated to be 1.1 F cm^−2^ based on the thickness of SiO_2_ (300 nm) and the lower BN layer (∼15 nm as determined by atomic force microscopy). With this information, we extracted the hole *μ*_F_ from the linear part of conductance at different temperatures (Fig. 2e,f). As shown in Fig. 2d, we achieved a high FET mobility *μ*_F_∼1,350 cm^2^V^−1^ s^−1^ and Hall mobility *μ*_H_∼790 cm^2^V^−1^ s^−1^ for Sample A at room temperature. Another four BN–BP–BN heterostructures also exhibit high mobility (∼800 cm^2^V^−1^ s^−1^) at room temperature (summarized in Supplementary Table 1). One possible reason for the high mobility in our BP samples is because their transport directions are nearly along the fast X-direction (see Fig. 1c) as confirmed by angle-dependent polarized Raman spectroscopy (see Supplementary Note 3 and Supplementary Fig. 4). The mobility along the X-direction can be two times larger than that along the Y-direction^13^,^15^. The transport-direction of Sample A is parallel to the X-direction. Although the mobility we achieved is still smaller than the values measured from bulk BP (∼2,000 cm^2^V^−1^ s^−1^)^33^, it is much larger than that observed in atomically thin MoS_2_ (refs 3, 4, 10) and few-layer BP supported on SiO_2_ (refs 12, 13, 14, 15). Theoretical calculations for few-layer BP^16^ indicate that a hole mobility over 4,000 cm^2^V^−1^ s^−1^ is achievable along the X-direction at room temperature. Hence, the quality improvement is still the critical limiting factor for the mobility of few-layer BP.

The high quality of the BN–BP–BN heterostructure is further confirmed by the observation of a high on–off ratio exceeding 10^5^ and insignificant hysteresis at room temperature (Fig. 2g), while previous work^14^ on BP supported on SiO_2_ showed a pronounced hysteresis (Δ*V*_g_∼80 V) depending on the sweep direction at room temperature, indicating the presence of a high density of charge traps. The small charge trap density in our BN–BP–BN heterostructure is due not only to the ultra-clean BN–BP interfaces but also to the high-temperature annealing (300–500 °C) of the encapsulated BP. As shown in Supplementary Fig. 2, in a BN–BP–BN heterostructure device without annealing, we observed a hysteresis Δ*V*_g_∼10 V, which is much smaller than that observed in BP supported on SiO_2_. Supplementary Note 4 and Supplementary Fig. 5 demonstrate the mobility changes of Sample D before and after annealing. The mobility of this sample measured at cryogenic and room temperatures indeed increases significantly. These results verified that high-temperature annealing can effectively suppress charge trap states and further improve the mobility and on–off ratio of BP devices.

The stability of the electrical performance of the BN–BP–BN heterostructure is examined under ambient conditions (with humidity around 60%). As shown in Fig. 2h, the mobility and on–off ratios show no degradation even after exposure to ambient conditions for a whole week. Obviously, the BN–BP–BN heterostructure completely hides the BP layer from ambient conditions and prevents it from reacting with O_2_-saturated H_2_O. With its high room temperature mobility, on–off ratios and stability, the BN–BP–BN heterostructure is a promising candidate for practical applications in electronic and optoelectronic devices.

At 1.7 K, the mobility of Sample A reaches ∼2,700 cm^2^V^−1^ s^−1^ (Fig. 2d) and the Hall mobility 
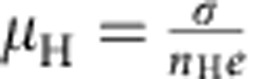
 is ∼1,500 cm^2^V^−1^ s^−1^ at large gate voltages, where *n*_H_ is the carrier density from Hall measurement, which is equal to the value extracted from the gate capacitance model *n*=*C*_g_(*V*_g_–*V*_th_)/*e* (see Supplementary Note 5 and Supplementary Fig. 6). The on–off ratios exceed 10^8^ at 1.7 K. The temperature dependence of the FET and Hall mobility of the BN–BP–BN heterostructures (Fig. 2e,f) shows a similar trend as reported previously for few-layer phosphorene^12^ and monolayer MoS_2_ (ref. 4). The mobility saturates at low temperatures (*T*<80 K), but follows the expression *μ*∼*T*^−*γ*^ at higher temperatures (*T*>80 K) due to phonon scattering. The extracted value *γ* is ∼0.57 for Sample A and ∼0.69 for Sample B (15 nm thick). These *γ* values are consistent with the ones observed in monolayer MoS_2_ covered with high-κ dielectric^4^ and few-layer phosphorene^12^, but are smaller than those found in bulk BP^34^ and other 2D materials^35^. Similar to the results reported previously^12^,^13^,^15^, the values of the Hall mobility measured from our samples are always smaller than that of their FET mobility (Supplementary Table 1). We believe that although charge traps are effectively suppressed in our BN–BP–BN heterostructures through annealing, other types of defects/impurities, such as vacancies and strain induced during the transfer process may still exist. These defects limit the mobility of few-layer BP particularly at low temperatures.

### Quantum oscillations in the sandwiched BP devices

**Figure 3:**
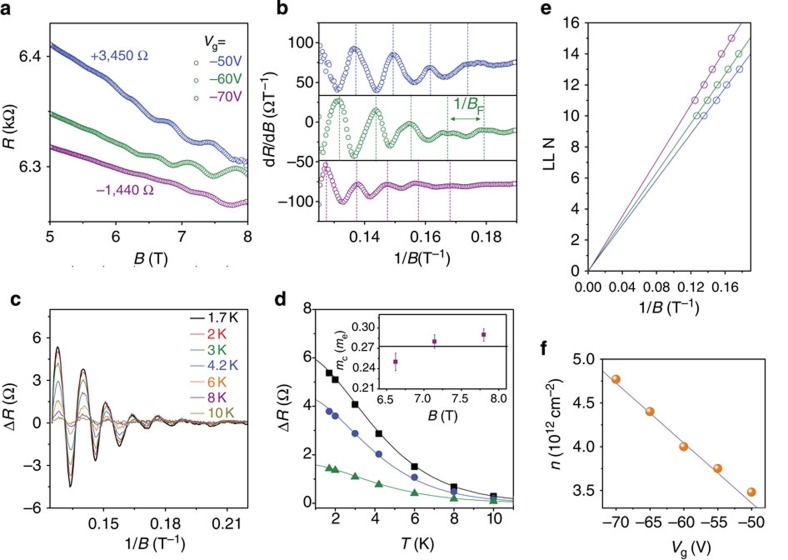
SdH oscillations in the 8-nm-thick BN–BP–BN heterostructure. (**a**) Resistance (*R*) plotted as a function of the magnetic field at gate voltages of −50 V (blue dots), −60 V (green dots) and −70 V (purple dots). (**b**) d*R*/d*B* plotted as a function of 1/*B*. The dashed lines indicate the oscillation period (increasing with gate voltages) of 1/*B*_F_. (**c**) Δ*R* plotted as a function of 1/*B* at *V*_g_=−60 V for different temperatures. (**d**) Fitting of the experimental results (dots) using the Lifshitz–Kosevich formula (solid line). The inset shows the fitting results of the cyclotron mass. (**e**) Landau diagram at different gate voltages that shows a Berry phase *β*=0. (**f**) The carrier densities determined from LL fitting (orange dots) and gate capacitance (purple line).

The quantum oscillations observed at magnetic fields *B*>6 T also attest to the high quality of BN–BP–BN heterostructures. Resistance at different gate voltages shows a negative magneto-resistance (Fig. 3a), indicating a weak localization effect^36^ previously observed in 2D electron gas systems containing disorder. In this kind of 2D electron gas systems, the time-reversal symmetry of the closed electron path is broken when a magnetic field is applied and the back scattering of electrons is suppressed resulting in a negative correction to the resistance. When *B*>6 T (corresponding to a mobility of ∼1/*B* near 1,670 cm^2^V^−1^ s^−1^), we start to see SdH oscillations at higher gate voltages where the Hall mobility becomes sufficiently high (Fig. 3a). The oscillation features are seen clearly in the form of d*R*/d*B* as shown in Fig. 3b (also see the data of Sample B in Supplementary Note 6 and Supplementary Figs 7 and 8).

The period of SdH oscillations^37^,^38^ in 2D electron gas is 1/*B*_*F*_ and the longitudinal resistance is given by Δ*R*=*R*(*B*,*T*)cos[2*π*(*B*_F_/*B*+1/2+*β*)], where *R*(*B*,*T*) is the amplitude and *β* (0≤*β*≤1) is the Berry phase. *β* is known to be 0 in conventional 2D electron gas systems and 1/2 in graphene^1^,^2^. To examine the 2D nature of hole gas in few-layer BP, we plot d*R*/d*B* as a function of 1/*B* for different gate voltages. As shown in Fig. 3b, the oscillations at different gate voltages are periodic but exhibit different frequencies. A smaller period 1/*B*_*F*_ is observed at higher gate voltages where the carrier density *n* is larger, which is consistent with the theoretical oscillation period^37^ expressed as 1/*B*_*F*_=2/Φ_0_*n*, where Φ_0_=4.14 × 10^−15^ Tm^2^ is the flux quantum. For example, at *V*_g_=−50 V the oscillation period is ∼0.0138 T^−1^, corresponding to a carrier density of ∼3.5 × 10^12^ cm^−2^, which is in excellent agreement with the value obtained from the gate capacitance model *n*=*C*_g_(*V*_g_−*V*_th_)/*e* (Fig. 3f). To further reveal the 2D nature of the charge carriers in few-layer BP, we carried out experiments to investigate the angular dependence of the SdH oscillations. The linear dependence between the magnetic field *B*_*F*_ and 1/cos *θ* confirms that the charge carriers in few-layer BP are in 2D nature, where *θ* is the tilting angle between the applied magnetic fields and the normal direction of the substrate plane (see Supplementary Note 7 and Supplementary Fig. 9). The Landau level (LL) index N is plotted and the linear fit yields a Berry phase *β*=0 in our BP samples (Fig. 3e). A smaller LL index would be achieved if magnetic fields could be further increased.

The cyclotron effective mass *m*_c_ of hole carriers in few-layer BP can be obtained by fitting the temperature dependence of resistivity Δ*R* (see Fig. 3c) using the Lifshitz–Kosevich formula *A*_T_∝*T*/sinh (2*π*^2^*k*_B_*Tm*_c_/*ℏeB*), where *k*_B_ is the Boltzmann constant. We fit Δ*R* at high magnetic fields to gain a better resolution as shown in Fig. 3d. The fitting results produce a cyclotron effective mass 0.27±0.02 *m*_e_ at *V*_g_=−60 V. Owing to structural anisotropy, the carrier effective mass in few-layer BP is also highly anisotropic. Previous theoretical analyses^16^ show that the effective masses along the X- and Y- directions are *m*_*x*_∼0.14 *m*_e_ and *m*_*y*_∼0.89 *m*_e_ respectively. The measured cyclotron effective mass in our BP samples is ∼0.27*m*_e_, which is reasonable as compared with the theoretical value 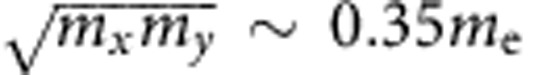 for few-layer BP^16^.

**Figure 4:**
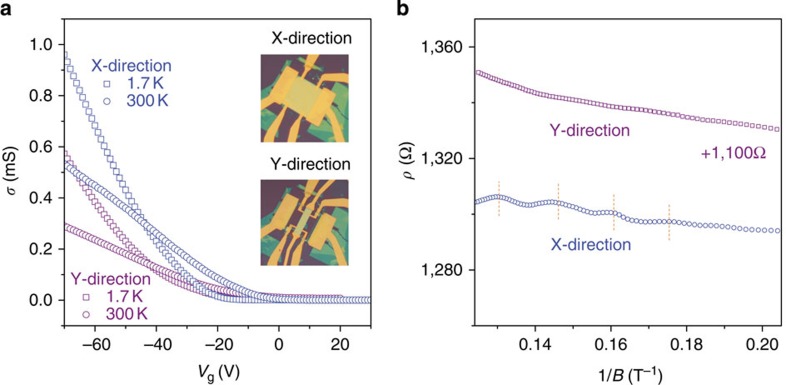
SdH oscillations in the BN–BP–BN heterostructure samples along the X- and Y- directions. (**a**) The conductivity of Sample C along the X- and Y- directions at 1.7 K and 300 K. The insets are optical images of Sample C before and after reshaping. (**b**) SdH oscillations measured at *V*_g_=−60 V from the samples along the X- and Y- directions.

Finally, we designed a special sample (Sample C, 10 nm thick) to demonstrate the effects of transport anisotropy on the SdH oscillations in BP. First, this sample was cut along the X-direction (Fig. 4a). The Hall mobility was ∼1,481 cm^2^V^−1^ s^−1^ at 1.7 K (∼716 cm^2^V^−1^ s^−1^ at room temperature). The SdH oscillations are clearly visible in Fig. 4b when the magnetic field is higher than 6 T. Then, the sample was reshaped along the Y-direction (see the inset in Fig. 4a). The Hall mobility changed to ∼820 cm^2^V^−1^ s^−1^ at 1.7 K (∼365 cm^2^V^−1^ s^−1^ at room temperature). This experimental results are consistent with the conclusions in previous reports^15^,^33^. However, the magneto-resistivity in this reshaped sample (along the Y-direction) did not show any signature of SdH oscillations with magnetic fields up to 8 T. A much stronger magnetic field is needed to observe SdH oscillations in the sample.

## Discussion

In summary, we have demonstrated high-quality BN–BP–BN heterostructures achieved by encapsulating atomically thin BP between BN layers followed by annealing at high temperature. The BN–BP–BN heterostructure protects BP from property degradation and allows us to investigate the intrinsic properties of BP. The BN–BP–BN samples show excellent stability in the atmospheric environment with a high mobility ∼1,350 cm^2^V^−1^ s^−1^ and a large on–off ratio exceeding 10^5^. Quantum oscillations and zero Berry phase are observed in BP hole gas at a magnetic field of 6 T at cryogenic temperatures. The ultra-clean interfaces realized by our fabrication process can effectively suppress charge trap states. They shed important light on how to improve the quality of atomically thin BP for practical applications in BP-based nanoelectronic devices.

## Methods

### Sample preparation and transport measurements

Bulk phosphorus and BN crystals (Polartherm grade PT110) were purchased from Smart-elements and Momentive, respectively. All atomically thin flakes were mechanically exfoliated by the scotch-tape microcleavage method. The thickness of BN and BP flakes was determined using an atomic force microscope (Veeco-Innova). The BN–BP–BN heterostructure was fabricated using the polymer-free van der Waals transfer technique^32^. The encapsulated BP was annealed in an argon atmosphere at 300–500 °C for 8 h before the upper BN layer was etched. No further annealing process was needed after deposition of the electrodes. Electrical measurements were performed using lock-in techniques in a cryogenic system (1.7–300 K and magnetic field up to 8 T).

## Additional information

**How to cite this article:** Chen, X. *et al.* High quality sandwiched black phosphorus heterostructure and its quantum oscillations. *Nat. Commun.* 6:7315 doi: 10.1038/ncomms8315 (2015).

## Supplementary Material

Supplementary Information Supplementary Figures 1-9, Supplementary Tables 1, Supplementary Notes 1-7 and Supplementary References.
