# Supplementary material for: High-quality sandwiched black phosphorus heterostructure and its quantum oscillations
Source: Nat Commun. 2015 Jun 23;6:7315. doi: 10.1038/ncomms8315 (PMC4557360; doi:10.1038/ncomms8315)
Supplement: Supplementary Information — Supplementary Figures 1-9, Supplementary Tables 1, Supplementary Notes 1-7 and Supplementary References. [file ncomms8315-s1.pdf]

Supplementary Figures

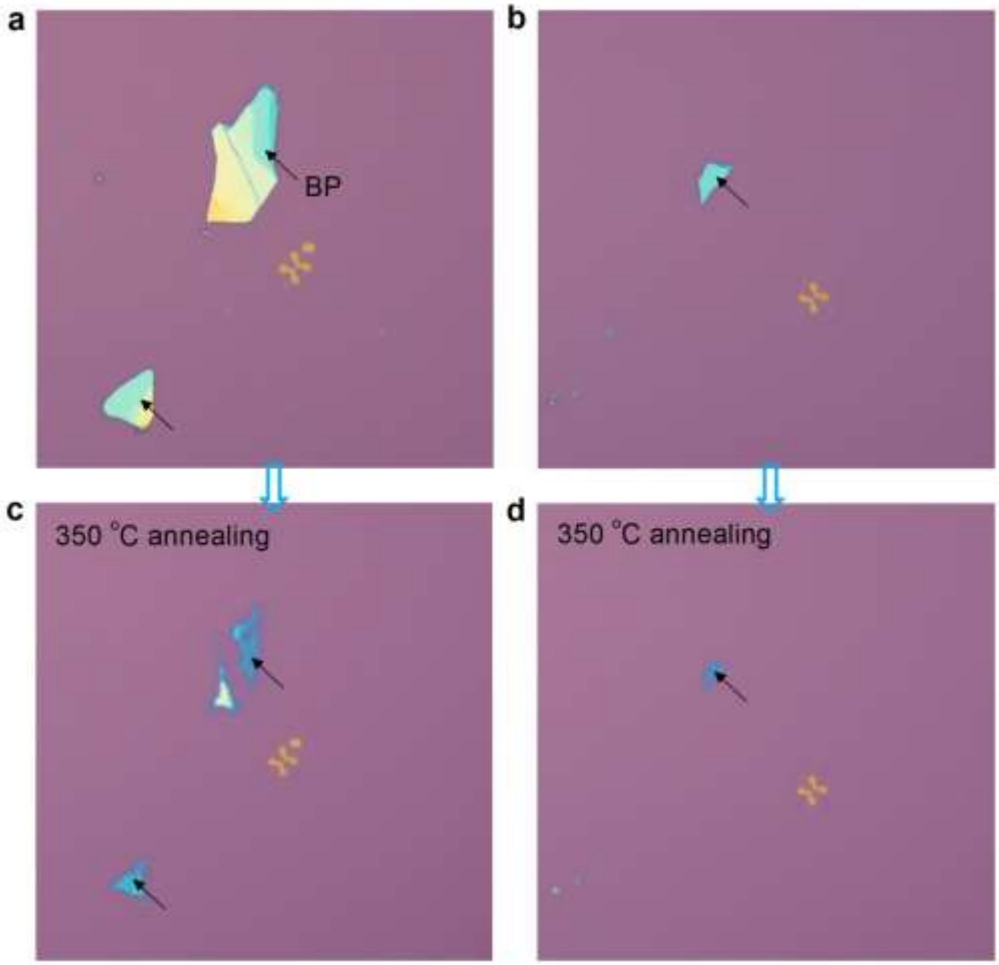

Supplementary Figure 1 | High-temperature annealing of BP flakes on SiO_2. (**a-d**) The optical images of three BP flakes on a SiO_2 substrate before (**a**,**b**) and after annealing (**c**,**d**) at 350 °C for 8 hours in argon atmosphere.

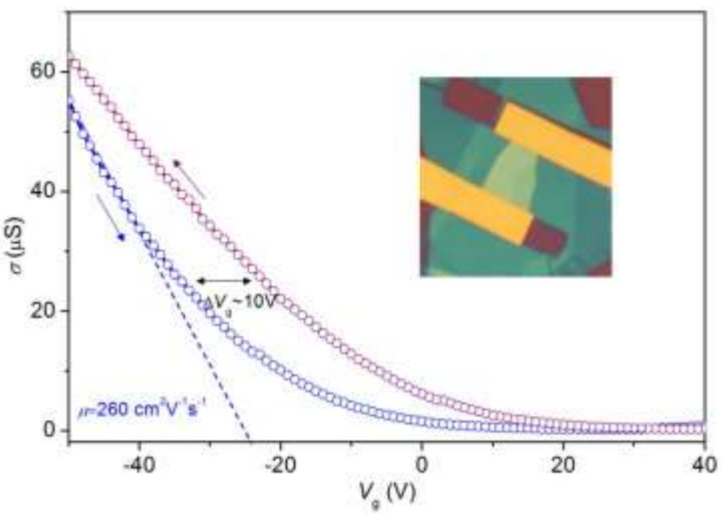

Supplementary Figure 2 | The hysteresis effects in the BN-BP-BN heterostructure without annealing. Without annealing treatment, the conductivity of a 7.5nm-BP heterostructure shows a hysteresis of about ΔV_{g} ~10 V at 300 K.

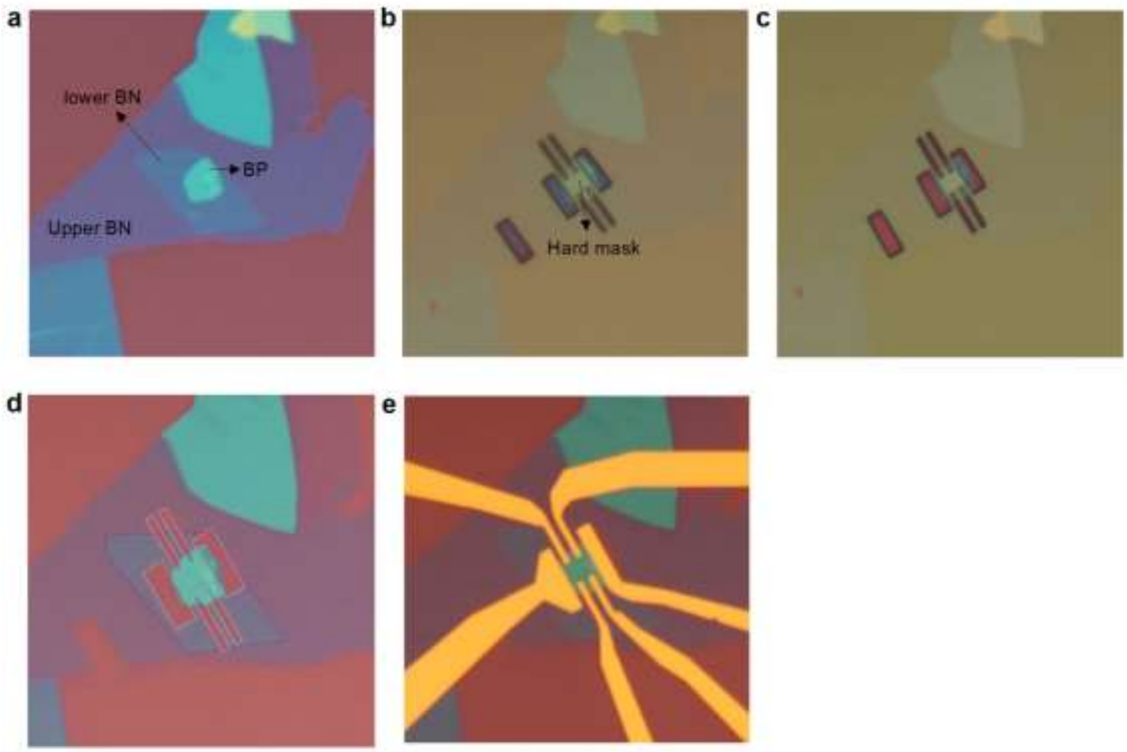

Supplementary Figure 3 | The process flow for fabrication of the BN-BP-BN heterostructure device. (**a**) Optical image of the BN-BP-BN heterostructure on SiO_2. (**b**) Defined areas of the hard mask for etching of BN layers. (**c**) BP layers remain after etching, while BN layers are etched away. (**d**) The BN-BP-BN heterostructure after removing the ZEP mask. (**e**) Optical image of a fabricated BN-BP-BN heterostructure Hall-bar device.

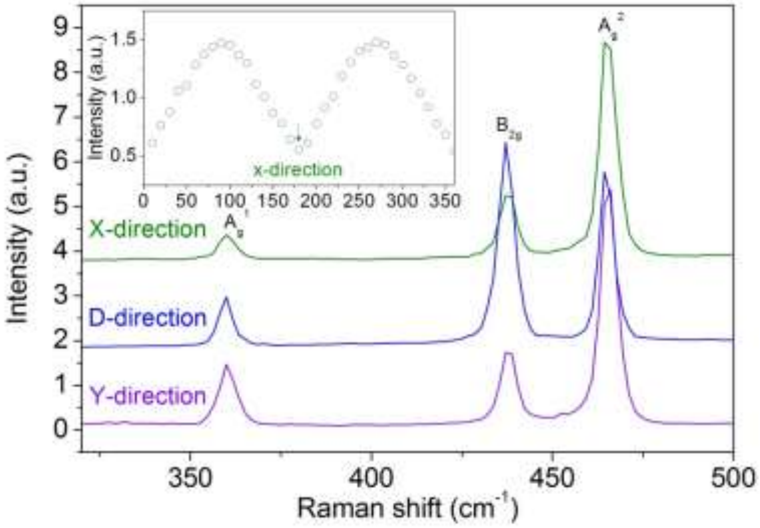

Supplementary Figure 4 | Polarized Raman spectra of BP layers. Polarized Raman spectra of BP layers along X-direction (green line), D-direction (blue line) and Y-direction (purple line). The incident laser wavelength is 532nm with linear polarization parallel to the BP sheets. The inset shows the intensity of A_g^1 at different directions. The intensity of A_g^1 reaches minimum along the X-direction and maximum along the Y-direction.

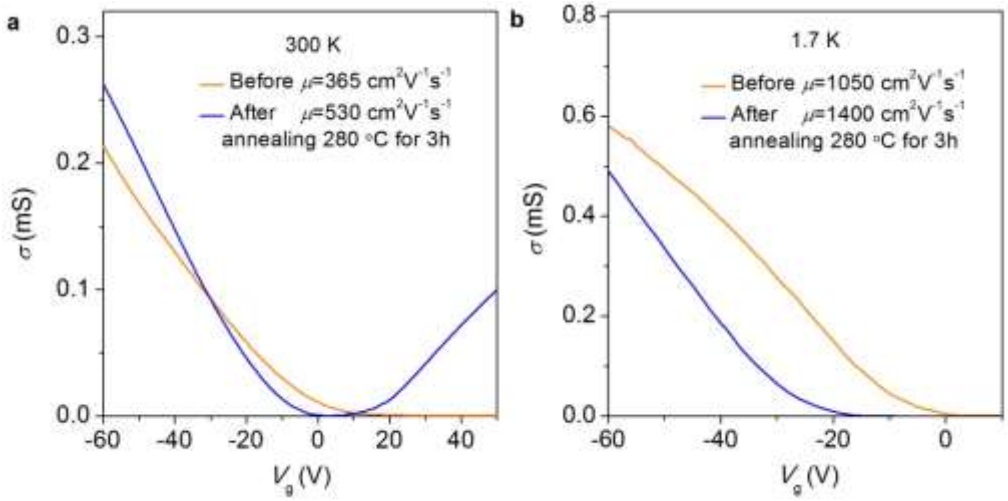

Supplementary Figure 5 | Annealing effects on the mobility of the BN-BP-BN heterostructure. (**a**,**b**) Conductivity of Sample D before (**a**) and after (**b**) annealing at 280°C for 3h in Ar atmosphere.

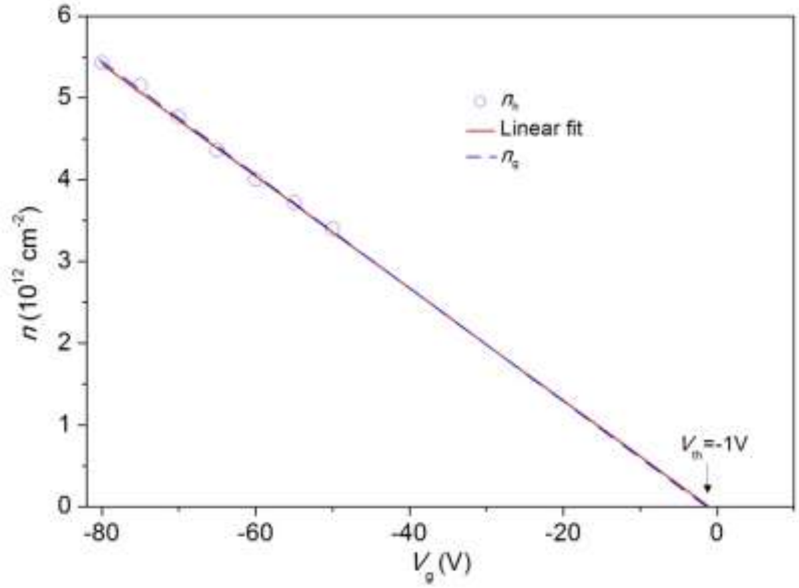

Supplementary Figure 6 | Determination of carrier densities from Hall measurements. The carrier density is determined from Hall measurements (blue dot) and gate capacitance (blue dashed line) at 1.7 K. The red line is the linear fit of n_h .

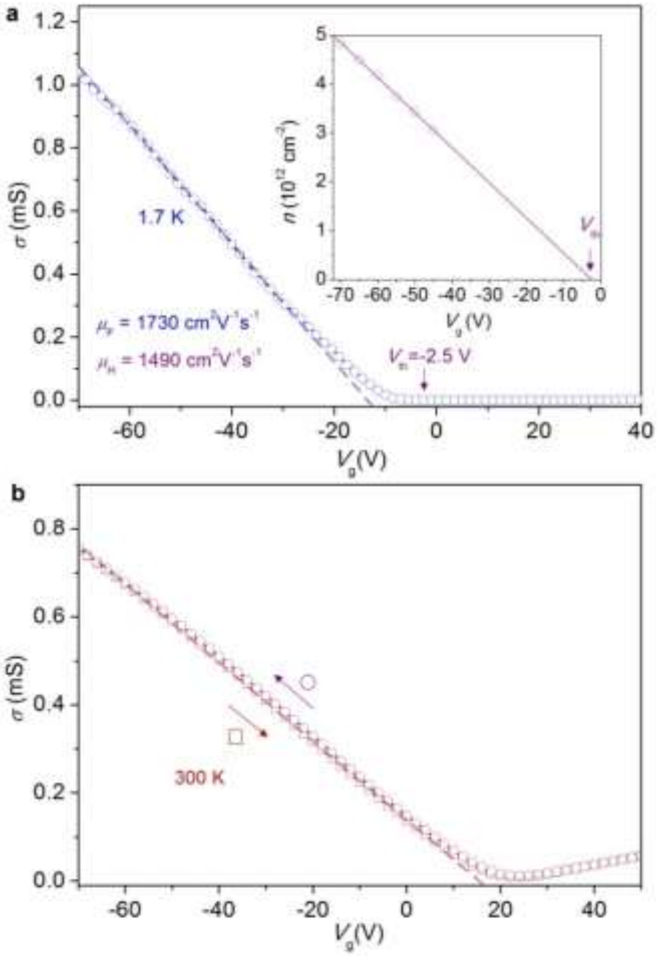

Supplementary Figure 7 | Estimation of the mobility and hysteresis in the 15nm-BP heterostructure. (**a**) The conductivity at 1.7 K with a field effect mobility $\sim 1730\ \mathrm{cm^2V^{-1}s^{-1}}$ and Hall mobility $\sim 1490\ \mathrm{cm^2V^{-1}s^{-1}}$. The inset shows the carrier density determined from Hall measurements. (**b**) The conductivity obtained under different gate sweeping directions, showing no hysteresis at 300 K.

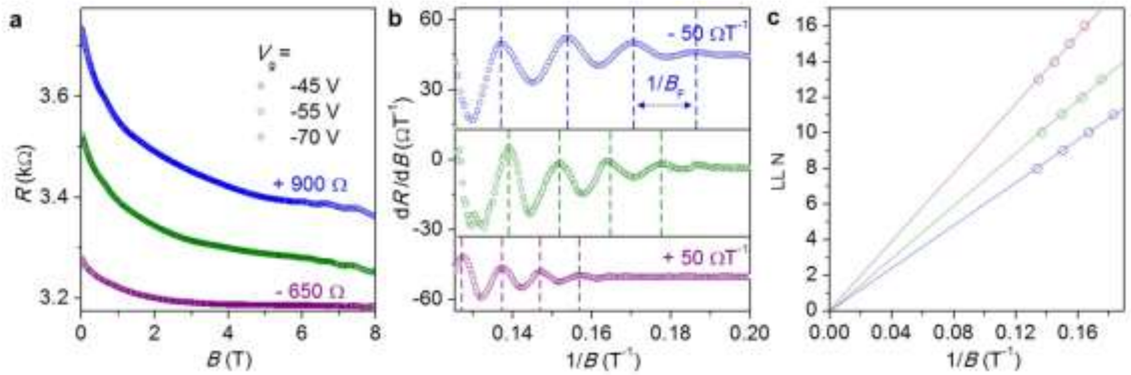

Supplementary Figure 8 | SdH oscillations in the 15nm-BP heterostructure. (**a**) The measured resistance of the 15 nm-BP sample plotted as a function of magnetic fields at -45 V (blue dots), -55 V (green dots) and -70 V (purple dots) gate voltages respectively. (**b**) $\mathrm{d}R/\mathrm{d}B$ plotted as a function of $1/B$ yields an oscillation period in $1/B_{\mathrm{F}}$ which becomes smaller at a higher gate voltage. (**c**) Landau diagram at different gate voltages.

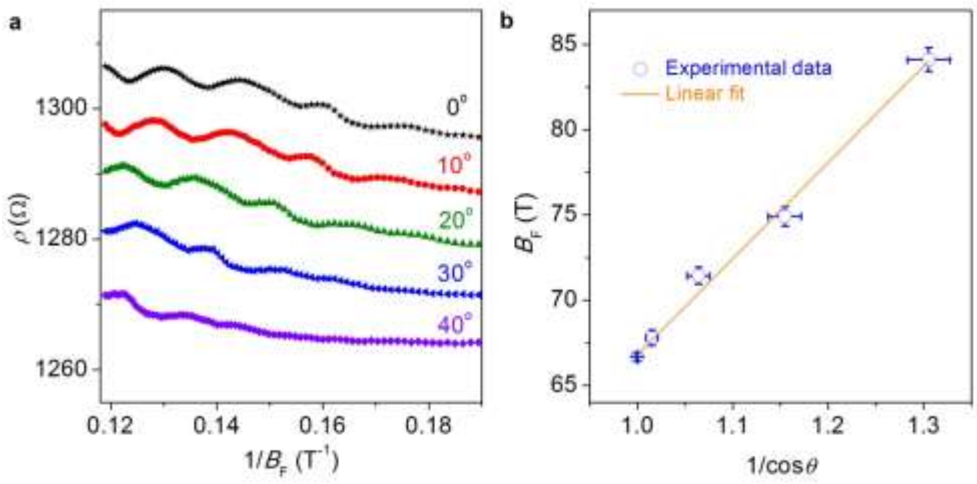

Supplementary Figure 9 | Angular dependence of SdH oscillations in few-layer BP. (**a**) Angular dependence of SdH oscillations of Sample C obtained at V_g=-60 V. (**b**) Experimental data of B_F dependence of the tilt angle θ (blue dots). The orange line shows the linear fitting.

Supplementary Tables

Supplementary Table 1. Information of BN-BP-BN heterostructure devices.

Sample No.	Transport direction	d nm	L μm	W μm	μ_{FET} 2K $cm^2V^{-1}s^{-1}$	μ_{FET} 300K $cm^2V^{-1}s^{-1}$	μ_h 2K $cm^2V^{-1}s^{-1}$	μ_h 300K $cm^2V^{-1}s^{-1}$
A	X-direction	8	16	3	2700	1350	1500	790
A (2-terminal)	X-direction	8	16	3	1386	516		
B	~20° off X	15	15	4.3	1730	850	1490	785
C	X-direction	10	19	22	2416	875	1481	716
C	Y-direction	10	21	5	1647	495	820	365
D (as prepared)	~20° off X	12	12	8	1050	365	788	261
D (annealed 280°C)	~20° off X	12	12	8	1400	530	1004	406
E	~18° off X	11	11.8	7.5	1653	675	1217	623
F	~40° off X	6	14	13.3	1546	626	925	510
G	~23° off X	13	13	8.5	1568	722	1230	650
H	X-direction	11	25	12	1852	865	1310	720
I (2-terminal)	~30° off Y	7.5	10	7.9	535	260		

Supplementary Notes

Supplementary Note 1

High-temperature annealing of BP flakes

The high temperature annealing ($300\ ^\circ C - 500\ ^\circ C$) of the entirely encapsulated BP flakes in argon (Ar) atmosphere before etching the upper BN layers can further improve the performance and quality of BP. As shown in Fig. 2c in the main text, the conductance of the 8nm-BP heterostructure shows no hysteresis at room temperature after annealing in Ar atmosphere at $350\ ^\circ C$ for 8 hours. The 15nm-BP heterostructure shown in the main text is annealed at $400\ ^\circ C$ for 8 hours and also shows no hysteresis at room temperature (Supplementary Figure 7b). The conductance of a 7.5nm-BP heterostructure (Sample I in Supplementary Table 1) without annealing shows a hysteresis $\Delta V_g \sim 10$ V at room temperature (Supplementary Figure 2), although it is much smaller than the value observed in BP on SiO_2[1]. The observed hysteresis is due to the charge trapping effect (showing a positive direction of the hysteresis) instead of the capacitive coupling to BP as discussed in Ref. 2. Obviously, the high-temperature annealing for the encapsulated BP can effectively suppress the charge trapping effect and improve the performance of the BN-BP-BN heterostructures. However, without covering BN layers, thin BP layers prepared on SiO_2 were quickly destroyed by annealing at high temperatures. As shown in Supplementary Figure 1, three BP samples are destroyed after annealing at $350\ ^\circ C$ for 8 hours.

Supplementary Note 2

The process flow for making BN-BP-BN heterostructures

To obtain an ultraclean BN-BP interface, the polymer-free van der Waals transfer technique[3] is adopted to pick up BP flakes by BN. Detailed process for this transfer technique has been reported in Ref. 3. Therefore, we demonstrate here the processes after assembling and transferring BN-BP-BN heterostructures onto SiO_2/Si substrates as shown in Supplementary Figure 3a.

To make a hard mask, 600 nm e-beam resist ZEP layers are prepared and then standard e-beam lithography is used to pattern the ZEP layers as shown in Supplementary Figure 3b. The exposed areas are etched by O_2-plasma. Since O_2-plasma etching rates for BN and BP are different, the BN layer can be quickly etched away while the BP layer still survives as shown in Supplementary Figure 3c. The white dashed lines in Supplementary Figure 3d show the etched areas for electrode deposition after removing the hard mask. Supplementary Figure 3e shows the optical image of a Hall-bar device in BN-BP-BN configurations.

Supplementary Note 3

Angle-dependent polarized Raman spectroscopy of BP

The crystallographic orientation of BP is identified by the angle-dependent polarized Raman spectroscopy as shown in Supplementary Figure 4. Three pronounced peaks A_g^1 (365cm^{-1}), B_{2g} (437cm^{-1}) and A_g^2 (465cm^{-1}) are clearly detected, which are consistent with previous results[4]. The X- (fast) and Y- directions (slow) of BP can be characterized by analyzing the amplitude of A_g^1 peak, which reaches minimum in the X-direction and maximum in the Y-direction[4] (see the inset of Supplementary Figure 4). The transport-direction of the Sample A is along the X-direction and that of Sample B is ~20 degree deviation from the X-direction.

Supplementary Note 4

Annealing effect on the mobility of sandwiched BP devices

We performed further experiments on the annealing effects for the BN-BP-BN heterostructure. First, Sample D (~12nm) is fabricated without any annealing treatment, which shows a FET mobility ~365 $cm^2V^{-1}s^{-1}$ at 300 K and ~1050 cm^2 $V^{-1}s^{-1}$ at 1.7 K as shown in Supplementary Figure 5. Then, we annealed this sample in argon atmosphere at 280 ^oC for 3h to avoid degradation of the metal electrode contacts. After annealing, the mobility increases to ~530 cm^2 $V^{-1}s^{-1}$ at room temperature and ~1400 cm^2 $V^{-1}s^{-1}$ at 1.7K. Hence, the mobility of BP can be improved by annealing when measured at room and cryogenic temperatures.

Supplementary Note 5

Determination of carrier densities

The Hall mobility is calculated according to $\mu_{\mathrm{H}} = \frac{\sigma}{n_{\mathrm{h}} e}$, where $n_{\mathrm{h}} = 1/R_{\mathrm{H}}e$ is the carrier density determined from Hall measurements and $R_{\mathrm{H}} = \mathrm{d}R_{\mathrm{xy}}/\mathrm{d}B$ is the Hall coefficient. The extracted n_h at different gate voltages (blue dot) is shown in Supplementary Figure 6. The linear fit of n_h (red line) intercepts the x-axis at $V_{\mathrm{g}} = -1\ \mathrm{V}$, which shows excellent agreement with the value obtained from the gate capacitance $n_{\mathrm{g}} = C_{\mathrm{g}}(V_{\mathrm{g}} - V_{\mathrm{th}})/e$ (blue dashed line), where $C_{\mathrm{g}} = 1.1\ \mathrm{F\,cm^{-2}}$ and $V_{\mathrm{th}} = -1\ \mathrm{V}$.

Supplementary Note 6

The mobility, hysteresis and quantum oscillations in Sample B

At 1.7 K, the field effect mobility of Sample B (the 15nm-BP sample) reaches $\sim 1730\ \mathrm{cm^2V^{-1}s^{-1}}$ as shown in Supplementary Figure 7a. The Hall mobility is $\sim 1490\ \mathrm{cm^2V^{-1}s^{-1}}$ calculated by $\mu_{\mathrm{H}} = \frac{\sigma}{n_{\mathrm{h}}e}$ and n_{h} is determined by the Hall measurement (the inset in Supplementary Figure 7a). No hysteresis is observed in the 15nm-BP heterostructure at room temperature as demonstrated by the conductivity with different gate sweeping directions (Supplementary Figure 7b).

The variation of measured resistance under different magnetic fields in the 15nm-BP heterostructure is consistent with that observed in the 8nm-BP sample including the negative MR (Supplementary Figure 8a), clear oscillation period of $1/B_{\mathrm{F}}$ (Supplementary Figure 8b) and zero Berry phase (Supplementary Figure 8c).

Supplementary Note 7

Angular dependence of SdH oscillations in few-layer BP

We have performed further experiments on the angular dependence of the SdH oscillations for few-layer BP. Supplementary Figure 9a shows the shifted resistivity of Sample C (~10nm) as a function of oscillation period $1/B_{\mathrm{F}}$ at different tilt angles θ between B and the axis perpendicular to the substrate plane. The obtained B_{F} as a function of $1/\cos\theta$ shows a good linear dependence which indicates the 2D nature of carriers in few-layer BP (see Supplementary Figure 9b).

Supplementary References

1. Koenig, S.P., Doganov, R.A., Schmidt, H., Neto, A.H.C. & Ozyilmaz, B. Electric field effect in ultrathin black phosphorus. *Appl. Phys. Lett.* **104** (2014)

2. Wang, H.M., Wu, Y.H., Cong, C.X., Shang, J.Z. & Yu, T. Hysteresis of electronic transport in graphene transistors. *Acs Nano* **4**, 7221-7228 (2010).

3. Wang, L. et al. One-dimensional electrical contact to a two-dimensional material. *Science* **342**, 614-617 (2013)

4. Xia, F.N., Wang, H. & Jia, Y.C. Rediscovering black phosphorus as an anisotropic layered material for optoelectronics and electronics. *Nat. Commun.* **5**, 4458 (2014).
